# Extensor Pollicis Brevis tendon damage presenting as de Quervain’s disease following kettlebell training

**DOI:** 10.1186/2052-1847-5-13

**Published:** 2013-06-03

**Authors:** Karuppaiah Karthik, Charles William Carter-Esdale, Sanjay Vijayanathan, Tony Kochhar

**Affiliations:** 1 Upper limb unit, King's College Hospital, London SE59RS, UK; 2 Peninsula Medical School, Exeter, UK; 3 London Bridge Hospital, London SE1 2PR, UK

## Abstract

Kettlebell exercises are more efficient for an athlete to increase his or her muscle strength. However it carries the risk of injury especially in the beginners. A 39 year old gentleman came to our clinic with radial sided wrist pain following kettlebell exercises. Clinically patient had swelling and tenderness over the tendons in the first dorsal wrist compartment, besides Finklesten test was positive. Patient had a decreased excursion of the thumb when compared to the opposite side. Ultrasound/MRI scan revealed asymmetric thickening of the 1st compartment extensors extending from the base of the thumb to the wrist joint. Besides injury to the Extensor Pollicis Brevis (EPB) tendon by repetitive impact from kettlebell, leading to its split was identified. Detailed history showed that the injury might be due to off-centre handle holding during triceps strengthening exercises. Our report stresses the fact that kettlebell users should be taught about problems of off-center handle holding to avoid wrist injuries. Also, in Kettlebell users with De Quervains disease clinical and radiological evaluation should be done before steroid injection as this might lead to complete tendon rupture.

## Background

Kettlebell workouts are intended to increase strength, endurance, agility and balance, helping both the muscular and cardiovascular system with dynamic, total-body movements [1-3]. Proper use of this cast iron weight requires strength, coordination and lots of practice as using this exercise involves multiple joints and many muscle groups working together [2,3]. Injuries to wrist are common especially with the beginners due to direct impact over the wrist or due to off-center handling. Our report highlights the management of a case with Extensor Pollicis Brevis (EPB) tendon damage and de Quervain’s disease following kettlebell training, which were not previously reported with weight training injuries.

## Case presentation

A 39 year-old male came to our clinic with radial sided wrist pain for three months duration in his dominant right hand. The pain started few weeks after he started using kettlebell for weight training exercises. While doing the exercises the pain increased a lot, especially when doing the triceps jerk. The patient did the triceps jerk while standing with shoulder forward flexed to 170 degrees and weights in the hand. The patient tends to hold the handle off-centre and so the other end of the handle impacts the radial side of the wrist during elbow extension, when the wrist was dorsiflexed and radially deviated by the weight of kettlebell. The pain in the wrist prevented him from doing exercises, lifting weights and gripping. Besides, the pain also affected his software profession from using keyboard for long time. He is otherwise fit and healthy.

On examination patient had severe tenderness and swelling over the tendons of Extensor Pollicis Brevis and Abductor Pollicis Longus in the wrist. Besides marked loss of active excursion of the thumb is noted. Finkelstein’s test was positive suggesting de Quervain’s disease. There was no evidence of neurological or vascular deficit.

**Figure 1:**
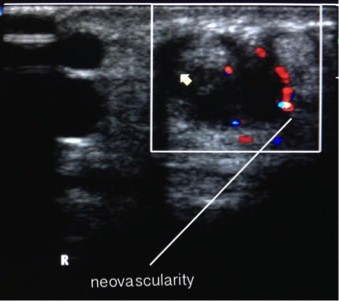
Marked hypervascularity along EPB tendon.

**Figure 2:**
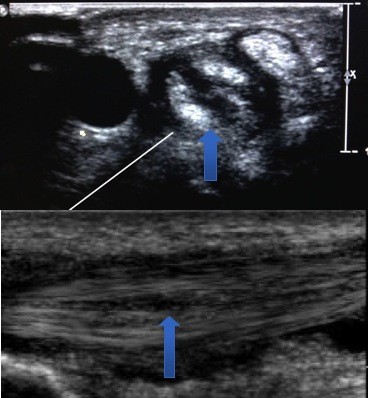
Both cross sectional (Top) and longitudinal (Bottom) view demonstrating EPB tendon split (Arrow).

**Figure 3:**
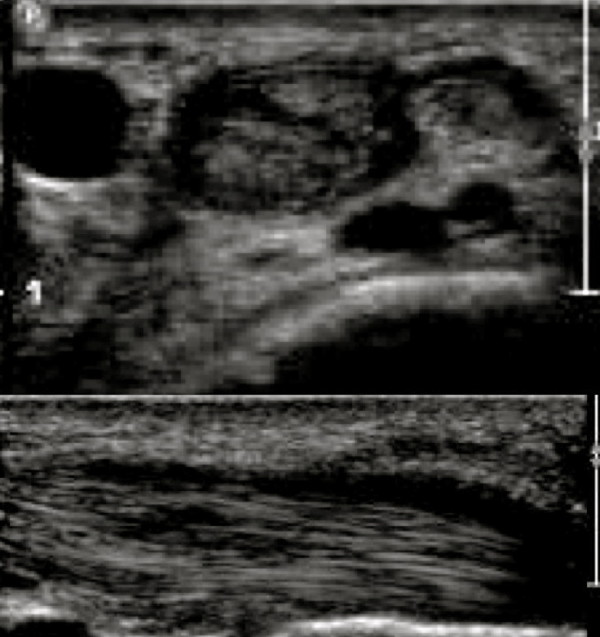
Three-month follow-up scan shows healing of EPB split, decreased vascularity and effusion.

The preliminary diagnosis was quite severe de Quervain’s tenosynovitis, as the patient had loss of thumb excursion an ultrasound was obtained to assess the integrity of the tendons. The steroid injection requested by the patient was withheld for further investigations. The X-Ray was normal with no evidence of arthritis or fracture. Ultrasound of the wrist showed thickened retinaculum appearing as marked hypoechogenic concentric material seen along the tendon of EPB. This was most marked in the wrist with associated hypervascularity (Figure 1) and effusion. A short segment along the carpal course of the EPB tendon had an interstitial split (Figure 2). In addition to this, the ultrasound scan also confirmed the clinical suspicion of de Quervain’s tenosynovitis. Patient accepted the fact that due to inexperience earlier in his training regime, the direct impacts during triceps jerk might have caused tendon injury. As the patient had tendon injury, the treatment plan was changed from steroid injection to wrist splint with thumb extension. The patient was reassessed after three weeks, as the patient was asymptomatic he returned back to his normal work. At three months follow-up the patient was asymptomatic and the repeat ultrasound showed healing of the EPB, absence of hypervascularity and resolution of effusion (Figure 3). The patient returned back to sports and fitness training using kettlebell.

## Discussion

Over the last few decades the incidence of emergency department visits due to injuries sustained by weight lifting has increased by 35% [4-6]. Wrist injuries specifically occur around half as much as those to the shoulder, with one study claiming around 12% of injuries from weight training to the upper limb [2]. However there seems to be no data on wrist injuries sustained due to exercise involving kettlebells as most of the injuries being categorised under the umbrella of weightlifting-related injuries.

Kettlebell exercises has become increasingly popular as they can be used to train both muculo-skeletal and cardio vascular system [1-3]. The unique design of the kettlebell and the exercises described with this may lead to an increase in the incidence of the injuries especially in beginners. Any pressing exercise is hard on the wrists if a kettlebell is involved, because of the off-center handle these exercises causes the wrists to get hyperextended by the force of the movement. Improper holding of the handle during exercise can lead to direct impact on the wrists as was in our patient.

De Quervain’s disease has been reported with sports and exercises related to the repeated use of wrist [7]. The patient presented with ultrasound features suggestive of type 2 de Quervain’s disease [8]. Treatment for this condition involves rest and/or steroid injection to reduce the inflammation around the tendons [9,10]. The disadvantage with the use of steroid injection is that it can cause detrimental effect on tendon healing and can even lead to tendon rupture [11,12]. As the patient had tendon injury the steroid injection without ultrasound may have lead to further complications.

## Conclusion

This case report insists the fact that Kettlebell users especially the beginners, should be taught about problems of off-center handle holding to avoid wrist injuries. In these patients with clinical suspicion de Quervains disease, ultrasound evaluation should be done before steroid injection to rule out any tendon injuries.

## Consent

Informed consent was obtained from the patient for publication/presentation.

## Competing interests

The authors declare that they have no competing interests.

## Authors’ contributions

KK, C-ECW and VS have made substantial contributions in acquisition, interpretation and drafting the manuscript. KT have critically revised and given final approval of the version to be published. All authors read and approved the final manuscript.

## Pre-publication history

The pre-publication history for this paper can be accessed here:

http://www.biomedcentral.com/2052-1847/5/13/prepub
